# Prenatal Dietary Exposure to Endocrine Disruptors and Its Lasting Impact on Offspring Health

**DOI:** 10.3390/toxics13100864

**Published:** 2025-10-11

**Authors:** Anastasios Potiris, Nikoletta Daponte, Efthalia Moustakli, Athanasios Zikopoulos, Eriketi Kokkosi, Nefeli Arkouli, Ismini Anagnostaki, Aikaterini Lydia Vogiatzoglou, Maria Tzeli, Angeliki Sarella, Ekaterini Domali, Sofoklis Stavros

**Affiliations:** 1 Third Department of Obstetrics and Gynecology, University General Hospital “ATTIKON”, Medical School, National and Kapodistrian University of Athens, 12462 Athens, Greece; vogiatzoglou.lydia@gmail.com (A.L.V.); sfstavrou@med.uoa.gr (S.S.); 2 Department of Obstetrics and Gynaecology, Medical School, University of Thessaly, 41500 Larissa, Greece; nikolettadaponte@gmail.com; 3 Department of Nursing, School of Health Sciences, University of Ioannina, 4th kilometer National Highway Str. Ioannina-Athens, 45500 Ioannina, Greece; ef.moustakli@uoi.gr; 4 Torbay and South Devon NHS Foundation Trust Lowes Brg, Torquay TQ2 7AA, UK; thanzik92@gmail.com; 5 Department of Midwifery, Faculty of Health and Caring Sciences, University of West Attica, 12243 Athens, Greece; ekokkosi@uniwa.gr (E.K.); mtzeli@uniwa.gr (M.T.); asare@uniwa.gr (A.S.); 6 Department of Obstetrics and Gynecology, Tzanio Hospital, 18536 Piraeus, Greece; narkouli@windowslive.com; 7 Medical School, National and Kapodistrian University of Athens, 11528 Athens, Greece; isanagnostaki3@gmail.com; 8 First Department of Obstetrics and Gynecology, Alexandra Hospital, Medical School, National and Kapodistrian University of Athens, 11528 Athens, Greece; kdomali@yahoo.fr

**Keywords:** endocrine disruptors, prenatal exposure, maternal diet, developmental origins of health and disease (DOHaD), offspring health

## Abstract

Environmental stressors during the crucial period of fetal development can have a substantial impact on long-term health outcomes. A major concern is dietary exposure to endocrine-disrupting chemicals (EDCs), which can readily cross the placenta and disrupt fetal hormonal signaling and developmental programming. Examples of these chemicals include bisphenols, phthalates, pesticides, and persistent organic pollutants (POPs). Prenatal exposure to EDC has been associated with long-term effects in children, including immune disruption, metabolic dysregulation, impaired neurodevelopment, and reproductive alterations, as evidenced by human cohort studies and experimental models. Epigenetic reprogramming, direct interference with endocrine signaling, and oxidative stress (OS) are hypothesized pathways for these adverse consequences, which often combine to produce long-lasting physiological changes. This narrative review summarizes current research on maternal dietary exposure to EDCs during pregnancy, highlighting associations with adverse child health outcomes. It also discusses the growing evidence of transgenerational effects, the potential mechanisms linking prenatal exposure to long-term outcomes, and the importance of understanding the roles of timing, dosage, and chemical type. By highlighting the necessity of focused interventions to lower maternal EDC exposure and lessen threats to the health of offspring, the review concludes by discussing implications for future research, preventive measures, and public health policy.

## 1. Introduction

Pregnancy is a critical period in an individual’s life, during which exposure to environmental factors can have lasting health consequences [1]. In addition to influencing developmental programming, intrauterine circumstances may also increase the risk of chronic diseases in adulthood, according to the DOHaD theory [2,3]. Nutritional deficiencies, psychological stress, and environmental pollutants during this critical window have been linked to higher risks of obesity, type 2 diabetes, cardiovascular disease, neurodevelopmental disorders, and reproductive dysfunction in later life [4,5,6]. Unlike previous narrative reviews that provided a broad overview of prenatal EDC exposure and developmental outcomes, the current analysis specifically focuses on dietary exposure pathways, their mechanistic underpinnings, and their transgenerational effects [7]. It integrates mechanistic insights from experimental models with human epidemiological risk measures (OR, RR), considers mixture effects and environmentally relevant exposures, and emphasizes long-term offspring health outcomes [8]. Within the DOHaD paradigm, this comprehensive approach offers a revised synthesis that links exposure biomarkers, food, and long-term offspring health consequences, highlighting aspects not fully addressed in prior reviews [3].

Endocrine-disrupting chemicals (EDCs) have become an important contributor to disruptions in normal developmental pathways. Exogenous EDCs can perturb endogenous hormonal signaling, thereby affecting the endocrine regulation necessary for proper growth and differentiation [9,10]. Although pregnant women are exposed to these toxins through multiple environmental sources, diet represents one of the most significant and consistent pathways of exposure. The food supply has been shown to contain EDCs, including pesticide residues, phthalates, bisphenols, and persistent organic pollutants (POPs) [11]. For instance, phthalates can migrate from food packaging into fatty foods such as meat and dairy, while bisphenol A (BPA) and its derivatives leach from plastic containers and the epoxy linings of canned goods [12]. Although many countries have regulatory restrictions, pesticide residues, especially those of organophosphates and organochlorines, continue to be present on fruits, vegetables, and grains. Dioxins and polychlorinated biphenyls (PCBs) are examples of POPs that bioaccumulate in the food chain and are frequently consumed through meat, fish, and dairy products [13,14].

Many of these substances are lipophilic, which means that they can easily pass through the placental barrier and build up in the tissues of the mother, exposing the fetus directly at crucial developmental times [15]. Prenatal exposure may affect endocrine and metabolic processes, with some impacts perhaps manifesting later in life, according to evidence from human studies and experimental models [16]. While there is currently no evidence for such impacts in people, evidence from both epidemiological research and experimental models further shows that these developmental disturbances may be mediated through epigenetic processes, increasing the prospect of transgenerational transfer of risk [17,18].

Adding to these concerns, new research shows that dietary exposure to EDCs occurs worldwide, including in countries with strict food safety laws, where levels of bisphenols, phthalates, and pesticide residues can be detected [19]. The necessity for a targeted examination of maternal food intake is highlighted by the abundance of these chemicals as well as their ability to disrupt immunological, metabolic, hormonal, and neurodevelopmental pathways. Prenatal food is a particularly significant element for public health interventions because exposure timing during sensitive windows of fetal development is a crucial driver of long-term health outcomes [20].

It should be noted that associations do not always imply causality, and the majority of the human research cited in this study is observational. In contrast to single chemicals, real-world exposures take the form of complex mixes of numerous EDCs, which may result in additive or synergistic effects that are not observed in single-chemical research [21]. Furthermore, many mechanistic and animal studies employ dosages that are higher than those commonly found in human exposures; hence, dose–response relationships, appropriate controls, and environmental relevance should be considered when interpreting these findings [22]. The review highlights the advantages and disadvantages of the existing research while offering a thorough synthesis of dietary EDC exposure, mechanistic data, and long-term offspring health effects by taking these considerations into consideration up front.

Understanding the health effects of foodborne EDCs has become a top public health concern due to their prevalence and the developing fetus’s increased vulnerability. This narrative review summarizes results from animal experiments and human epidemiological research that have been published in peer-reviewed publications over the last 20 years, paying special attention to the effects of dietary EDC exposure during pregnancy on the health of the fetus. Instead of conducting a systematic study, this review critically synthesizes the most important data, mechanistic understandings, and policy considerations to help reduce the long-term health effects of endocrine disruptor exposure during pregnancy. To provide researchers, clinicians, and policymakers with a thorough understanding, this review also attempts to compare experimental and human data, identify potential risk mitigation techniques through dietary and regulatory measures, and highlight information gaps.

### Methodological Note

A systematic search approach was employed to find pertinent literature, despite the narrative aspect of this review. Combinations of the following terms were used to search PubMed, Scopus, and Web of Science databases for peer-reviewed research published between 2005 and 2025: “endocrine disruptors”, “prenatal exposure”, “maternal diet”, “offspring health”, “epigenetics”, “neurodevelopment”, “metabolic”, “reproductive” and “immune outcomes”. Recent experimental research, comprehensive reviews, and human epidemiological studies that specifically addressed prenatal dietary exposure to EDCs were preferred. To ensure comprehensiveness, important policy and toxicology papers were added to the references. The inclusion criteria were limited to English-language publications, and studies with statistically documented relationships and clearly defined exposure measurements (such as blood, urine, or dietary intake biomarkers) were given priority.

## 2. Prenatal Dietary Sources of Endocrine Disruptors

### 2.1. Bisphenols

Among the dietary endocrine disruptors that have been investigated the most are bisphenols, especially bisphenol A (BPA) [23,24]. These substances are utilized in the production of epoxy resins and polycarbonate plastics, which are frequently found in food containers, drink bottles, and the linings of canned goods. Consequently, they may infiltrate food and drink, particularly in hot or acidic environments [25]. Numerous food products have been found to contain BPA and its structural equivalents, bisphenol S (BPS) and bisphenol F (BPF), which have also been tested in human biological samples, such as maternal urine and, in certain investigations, amniotic fluid [26]. Research suggests that during crucial stages of development, BPA might directly expose the growing embryo by passing through the placental barrier. Birth weight, metabolic indices, and neurobehavioral outcomes in children have all been linked to changes in maternal urine BPA concentrations. For instance, 1.4-fold greater odds of lower birth weight and 1.6-fold higher chances of neurobehavioral changes in children were linked to maternal urine BPA concentrations in the top quartile (95% CI: 1.1–1.9) [27,28]. According to pooled analyses from recent meta-reviews, every doubling of maternal urinary BPA concentration is associated with a 0.2–0.3 kg decrease in birth weight when compared to the groups with the lowest exposure levels and raises the odds of negative neurobehavioral outcomes by roughly 25–35% (pooled OR = 1.3; 95% CI: 1.1–1.6) [27,28]. Early cohort results indicate similar relationships with changed birth size and behavioral indices (OR = 1.2–1.4; 95% CI: 1.0–1.8), notwithstanding the paucity of epidemiologic data on BPS and BPF [29,30]. Although many nations have implemented regulatory restrictions on BPA, the increasing use of substitute chemicals like BPS and BPF raises concerns regarding “regrettable substitutions”, particularly since these counterparts might have comparable endocrine-disrupting qualities [29,30].

### 2.2. Phthalates

Phthalates are another significant class of dietary EDCs that are frequently employed as plasticizers to make plastics more flexible in processing materials, storage equipment, and food packaging. These substances can readily seep into foods, especially those high in fat, such as meat, dairy products, and oils, because they are not chemically bonded to plastics [31,32]. The identification of phthalate metabolites in maternal urine across populations globally indicates that pregnant women are exposed to phthalates on a large scale.

Elevated maternal phthalate metabolite levels have been associated in epidemiological studies with impaired male offspring reproductive development, and other research has suggested links with a higher risk of childhood respiratory illnesses, such as asthma and wheeze [33]. For instance, according to cohort data, offspring who had maternal urine concentrations of di(2-ethylhexyl)phthalate (DEHP) metabolites in the highest exposure quartile were nearly 1.9 times more likely to have impaired male genital development (OR = 1.87; 95% CI: 1.12–3.12) and were twice as likely to wheeze (OR = 2.03; 95% CI: 1.15–3.57) [27,34]. According to similar results from other perspective studies, children of mothers who were exposed to high levels of phthalates during pregnancy had a 30–60% higher chance of developing asthma and rhinitis [29,35]. These conclusions are supported by experimental evidence indicating that phthalates may disrupt immune regulation, metabolic programming, and steroid hormone production. Phthalates can be anti-androgenic or estrogenic substances that mechanistically disrupt endocrine signaling, change gene expression through epigenetic alterations, and cause OS. These effects can impact multiple organ systems during critical fetal developmental stages [36].

Diet is the primary cause of exposure due to the pervasiveness of phthalate contamination in the food chain [36]. Prenatal exposure to phthalates is a major public health concern due to their widespread presence and ability to cross the placental barrier. Therefore, dietary modifications, safer packaging, and continuous monitoring of food phthalate levels are essential strategies to reduce fetal exposure and minimize long-term health risks.

### 2.3. Pesticides and Agricultural Chemicals

Particularly in areas with extensive agricultural operations, dietary exposure to pesticides is still a concern [37]. Despite regulatory limitations intended to prevent exposure, organophosphates, carbamates, and organochlorines are among the most often found groups of pesticide residues in fruits, vegetables, and grains [38]. These substances may expose the fetus during pregnancy due to their accumulation in maternal tissues and persistence in the food chain. Exposure to pesticides during pregnancy has been linked to lower birth weight, endocrine disruption, and neurocognitive impairments such as attention problems and reduced IQ [7]. Measurable neurodevelopmental effects have been linked to maternal exposure to organophosphate insecticides in large prospective birth cohorts. For instance, children exposed to the highest tertile of prenatal chlorpyrifos had a 5.5-point drop in IQ at age 7 (95 percent CI: 2.8–8.2) and 1.4-fold increased chances of attention-deficit symptoms [39]. The risk of cognitive delay is also increased by about 20–40% when prenatal organophosphate metabolite levels double (OR = 1.2–1.4; 95% CI: 1.05–1.8), according to pooled analyses of U.S. and European cohorts [40,41]. These quantitative results support causal inference by highlighting the consistency of relationships found in separate human research. Experimental studies indicate that even low-level gestational exposure can disrupt neural differentiation and endocrine signaling, while large birth cohort studies provide strong evidence linking maternal dietary pesticide exposure to adverse developmental outcomes [42].

In addition to cognitive effects, prenatal pesticide exposure has been linked to immunological and metabolic changes in the offspring, including insulin resistance, altered glucose metabolism, and increased susceptibility to infections and allergic diseases. Genetic vulnerability, co-exposure to other EDCs, and maternal nutrition are among the factors that may influence the risk. Overall, these findings suggest that dietary pesticide exposure remains a significant public health concern, particularly for pregnant women and developing fetuses, despite existing regulatory controls. They also highlight the necessity of ongoing monitoring and risk-reduction strategies.

### 2.4. Persistent Organic Pollutants (POPs)

PCBs, dioxins, and several banned pesticides are examples of POPs that remain a global concern due to their high lipophilicity and resistance to degradation. These substances are predominantly found in dietary sources such as meat, fish, and dairy products [13,43], where they accumulate in fatty animal tissues. Dairy products may also contain natural estrogens in addition to POPs, as pregnant cows are frequently milked, resulting in detectable quantities of estrogen in the milk [44].

Epidemiological research has connected prenatal exposure to POPs to several harmful health outcomes in children, including immunotoxicity, thyroid hormone abnormalities, and metabolic abnormalities that may increase the risk of obesity and diabetes in the future [45,46]. These associations have been supported by quantitative evidence from human cohorts. For example, according to data from the Norwegian Mother and Child Cohort (MoBa), children whose mothers were exposed to the highest quartile of PCBs during pregnancy were 1.5 times more likely to be overweight or obese at age 5 (RR = 1.52; 95% CI: 1.12–2.07) [45]. Similarly, a meta-analysis of 21 studies found that the risk of childhood obesity increased by 22% for every doubling of maternal serum POP concentrations (pooled OR = 1.22; 95% CI: 1.10–1.36) [46]. Other research has shown that neonates with increased prenatal dioxin or PCB exposure are 1.4–1.6 times more likely to have changed thyroid hormone levels and immunological biomarkers [47].

Mechanistic studies suggest that even minimal prenatal exposure to POPs and dietary estrogens may have long-lasting impacts on a child’s health. Long-term physiological changes may result from POPs’ disruption of lipid and glucose metabolism, interference with endocrine signaling, modification of epigenetic programming, and induction of OS, according to mechanistic studies. While their environmental stability permits exposure to continue long after their usage has stopped, their prolonged biological half-lives raise the possibility of cumulative and transgenerational effects [48].

Thus, in addition to being a route of exposure in the present, the dietary channel also bears the legacy of previous industrial and agricultural practices. Regulations to prevent exposure, promotion of dietary advice to reduce exposure, and ongoing monitoring of POP levels in food. Table 1 provides an overview of common EDC classes, their maternal exposure evidence, and reported health effects on offspring [35,36,37,38,39,40,41,42].

## 3. Mechanistic Insights

Interpreting the various results seen in both human and experimental investigations require an understanding of the processes by which prenatal dietary exposure to EDCs impacts the health of the offspring. Current research identifies many interconnected processes, including placental failure, OS and inflammation, disruption of hormonal signaling, and epigenetic reprogramming [7,51,52]. Together, these processes form a complex web of interactions that shape fetal development and may produce lasting health consequences throughout the life course and, in some cases, across generations. Notably, in real-world situations, prenatal exposure usually involves combinations of several EDCs rather than individual compounds. Thus, the following molecular pathways—hormonal disruption, oxidative stress, placental malfunction, and epigenetic reprogramming—may be impacted by synergistic or combined effects that differ from those reported from single-chemical experimental investigations [16,53].

### 3.1. Epigenetic Reprogramming

Epigenetic reprogramming, which causes heritable modifications in gene expression without changing the underlying DNA sequence, is one of the main ways that EDCs may have long-lasting impacts [54,55]. Experimental models have demonstrated that EDCs like BPA, phthalates, and certain pesticides can cause DNA methylation, histone modifications, and changes in the production of non-coding RNA during crucial stages of fetal development. These changes have the potential to last long after pregnancy, successfully “programming” patterns of gene expression that impact vital biological processes [56,57].

Such epigenetic modifications have far-reaching downstream effects that may modify gene networks that control immune system regulation, neurodevelopmental processes, metabolic balance, and reproductive health [47,48]. Despite the lack of direct human data, experimental research indicates that some of these epigenetic changes might be transgenerational—that is, they could be passed on to future generations without further exposure. This hypothesis presents serious concerns since it suggests that dietary exposure to EDCs during pregnancy may have an impact on future generations’ health in addition to the immediate children [49,50].

### 3.2. Hormonal Signaling Disruption

Through imitating, inhibiting, or altering endogenous hormones, EDCs can disrupt the endocrine system’s regular operation. POPs may change thyroid hormone homeostasis, while substances like phthalates and bisphenols might have estrogenic or anti-androgenic effects [19,58]. Hormonal transmission is essential for coordinating organogenesis, brain development, and the development of the reproductive system during the prenatal stage, which makes these abnormalities especially dangerous [59].

Because hormones act as precise molecular signals that guide developmental programming, interference during this sensitive window may result in long-lasting or even permanent alterations [60]. Consequences can manifest across multiple domains, including impaired growth trajectories, abnormal neurobehavioral programming, altered stress responses, and disturbances in reproductive maturation. By dysregulating key pathways such as estrogen, androgen, thyroid, and glucocorticoid signaling, prenatal EDC exposure may predispose offspring to a spectrum of adverse outcomes that can persist into adolescence and adulthood [13,53].

### 3.3. OS and Inflammation

Increased OS and inflammatory reactions in both maternal and fetal tissues have also been linked to prenatal exposure to EDCs [61,62]. Experimental evidence demonstrates that BPA and several pesticides can raise levels of pro-inflammatory cytokines and reactive oxygen species (ROS) in the placenta and fetal organs, which may contribute to cellular injury, impaired organogenesis, and modified metabolic programming [63,64]. Chronic OS during development can disrupt multiple organ systems, contributing to outcomes such as poor neurodevelopment, insulin resistance, and cardiovascular alterations in offspring [65,66]. Crucially, inflammation and OS frequently work in tandem with other processes like placental malfunction and hormone imbalance. The systemic character of EDC consequences on the growing organism is shown by this interaction, which makes it challenging to separate individual effects.

### 3.4. Placental Dysfunction

EDCs may affect the placenta’s structure and function, which is crucial for fetal growth and nutrient delivery. Endocrine signaling abnormalities, reduced nutrition transport, and altered placental vascularization have all been associated with exposure to bisphenols, phthalates, and POPs across observational and experimental studies [7,23]. These placental alterations may limit the fetus’s access to oxygen and nutrition, which could affect growth patterns, thus setting the newborn up for long-term health issues, metabolic disorders, and low birth weight [67,68]. The capacity of EDCs to influence placental efficiency raises the possibility of long-lasting physiological and metabolic impacts, which may have both direct and indirect effects on fetal development [69]. Since the placenta is the vital link between the surroundings of the mother and the fetus, its disturbance can directly threaten the fetus as well as exacerbate other harmful processes like hormonal imbalance and OS. Because of this, a key node in the molecular pathways of prenatal EDC exposure is placental malfunction [70].

## 4. Offspring Health Outcomes

In offspring, prenatal exposure to EDCs has been associated with potential adverse effects on immune function, metabolism, reproduction, and neurodevelopment. Depending on the kind, timing, and amount of EDC exposure during pregnancy, these effects can persist throughout adolescence and adulthood [71,72]. The key offspring health outcomes associated with prenatal EDC exposure are summarized in Table 2.

### 4.1. Neurodevelopment

Neurodevelopment is particularly vulnerable to prenatal exposure to EDCs because the fetal brain’s development depends on tightly regulated hormonal and communication networks [79]. Human cohort studies have found that maternal exposure to bisphenols and phthalates is associated with behavioral outcomes such as attention-deficit/hyperactivity disorder (ADHD), traits associated with autism spectrum disorders, and cognitive performance declines, including lower IQ scores [73,80]. These findings are supported by experimental animal models indicating that prenatal BPA exposure can alter neuronal development, synapse formation, and neurotransmitter systems. These findings suggest that EDCs may influence critical neurodevelopmental processes through epigenetic reprogramming, OS, and hormonal disruption [81,82,83]. Research indicates that long-term cognitive and behavioral trajectories can be changed by even slight disturbances during critical phases of brain development. These results highlight the possibility that prenatal EDC exposure may be a factor in the rising prevalence of neuropsychiatric and cognitive impairments seen in contemporary societies, given that neurodevelopmental abnormalities can last a lifetime.

In addition to cognitive and behavioral outcomes, prenatal EDC exposure may also have lasting effects on reproductive function and social behaviors. According to research on humans and animal models, EDCs can interfere with the limbic and hypothalamic areas’ development, changing later-life social interactions, fertility, and sexual maturation [84,85,86]. These effects likely involve changes in hormone signaling, neural circuitry, and epigenetic programming, further emphasizing the critical vulnerability of the developing brain to even subtle endocrine disturbances.

### 4.2. Metabolic Health

Maternal dietary exposure to EDCs has been linked to alterations in offspring metabolic programming. Evidence suggests that prenatal exposure may increase the risk of obesity, insulin resistance, and metabolic syndrome in both childhood and adulthood [87,88]. These effects could be caused by changes in the formation of pancreatic β-cells, alterations in the endocrine signaling pathways that govern hunger and fat deposition, and epigenetic changes in the genes that control energy metabolism, according to mechanistic studies. These findings suggest a potential link between prenatal EDC exposure and the global increase in obesity and metabolic disorders [9,89,90]. Given that metabolic disorders are currently the largest cause of illness and mortality worldwide, this connection is particularly troubling from the standpoint of public health. Interventions focusing on maternal diet and exposure reduction during pregnancy may be effective strategies for disease prevention at the population level if prenatal exposures can predispose people to obesity and type 2 diabetes [91,92,93].

### 4.3. Reproductive Outcomes

Prenatal EDC exposure also affects the reproductive system. In animal studies, male offspring of BPA-exposed mothers exhibited lower sperm counts, disrupted testicular architecture, and reduced sperm motility [94]. In females, prenatal exposure has been associated with altered ovarian follicle development, disrupted estrous cycles, and changes in hormone levels [95,96]. Human research, however limited, indicates that maternal exposure to EDC may be linked to changes in the timing of pubertal development and the levels of reproductive hormones in offspring. These results demonstrate how early-life endocrine disturbance may affect fertility and reproductive health in the long run [97,98]. These findings have wide-ranging societal implications since reproductive health influences both population-level fertility patterns and individual well-being. Reduced ovarian reserve, decreased sperm quality, and altered puberty timing may be factors in the increased incidence of infertility and subfertility, supporting the notion that dangers to reproductive health start much younger than previously thought [99,100].

### 4.4. Immune System Changes

Prenatal exposure to EDCs, particularly POPs, may alter the function of the offspring’s immune system. According to research, children exposed to EDCs may exhibit weaker vaccine responses, increased susceptibility to infections, and higher rates of allergic conditions, including eczema and asthma [79,101]. Mechanistic evidence suggests that EDCs may affect cytokine synthesis, immune cell differentiation, and inflammatory pathways through OS and epigenetic modifications. These early immunological alterations may impact an individual’s susceptibility to disease in the future and have long-term consequences [102,103,104]. Prenatal exposures might weaken immunological resilience, making children more susceptible to infections and non-communicable diseases, according to this line of data. Since the early years of life are crucial for the development of the immune system, perturbations at this time may lay the groundwork for autoimmune diseases, chronic inflammation, and compromised host defenses that persist into adulthood [105].

## 5. Public Health Implications

The prevalence of EDCs in the food supply emphasizes how crucial preventive measures are to safeguarding the health of both the mother and the fetus [106,107]. Modern societies make dietary exposure virtually inevitable, therefore public health interventions should take a multifaceted strategy, encompassing behavioral, educational, and regulatory measures [108].

Stricter regulation of chemicals used in food processing, storage, and packaging is crucial at the regulatory level. Reducing the transfer of EDCs into food products can be achieved through policies that restrict the use of phthalates, bisphenols, and other potentially hazardous additives [12,109]. Improved monitoring of POPs and pesticide residues in agricultural products can reduce prenatal exposure and assist in guaranteeing adherence to safety regulations. Since POPs remain in the environment and bioaccumulate in the food chain across national boundaries, international cooperation may be especially beneficial for their regulation [43,110].

Campaigns for public education are also essential. It is important to educate women of reproductive age and pregnant women about the sources of dietary EDCs and doable ways to limit exposure [111,112]. Recommendations include consuming fresh, minimally processed foods; avoiding the use of plastic containers to heat or store fatty foods; maintaining a varied diet to reduce the accumulation of specific toxins; and limiting the intake of canned or packaged products [113,114]. During delicate stages of fetal development, educational interventions might decrease cumulative exposure and enable consumers to make educated selections [115].

Research-based guidelines should be developed to support medical professionals in advising patients on environmental exposures and prenatal nutrition. Beyond the standard recommendations for maternal nutrition and lifestyle, incorporating EDC risk information into prenatal care programs may help ensure that expectant mothers receive evidence-based advice on minimizing exposure [116,117].

Reducing dietary EDC exposure during pregnancy requires a multifaceted strategy that combines clinical guidance, public education, and regulatory action. Such initiatives could support the long-term health of the next generation and reduce the risk of adverse developmental outcomes [118,119,120]. Table 3 outlines key strategies, their targets, and potential benefits for minimizing maternal dietary exposure to EDCs [4,97,100,101,102].

## 6. Conclusions

The health of the offspring may be at risk due to prenatal dietary exposure to EDCs, which can impact immunological function, metabolism, neurodevelopment, and reproduction. Even low-level, prolonged exposure can affect prenatal programming, cause epigenetic alterations, and disrupt hormonal signaling, according to evidence from human and experimental studies. These effects may sometimes last into maturity or across generations.

There are still significant gaps in spite of these discoveries. Long-term effects, dose–response relationships at doses that are important to the environment, and the cumulative effects of chemical combinations are not well understood. Additional research is also needed to examine interactions with maternal nutrition, lifestyle, and other environmental factors.

Multidisciplinary research, as well as aggressive public health and regulatory actions, will be needed to address these issues. Protecting vulnerable groups and promoting healthier developmental paths for future generations may be possible by lowering prenatal dietary EDC exposure through stronger chemical restrictions, improved monitoring, and consumer education.

## Figures and Tables

**Table 1:** Major dietary sources of EDCs during pregnancy and associated offspring health outcomes.

Class of EDCs	Common Dietary Sources	Evidence of Maternal Exposure	Reported Offspring Health Outcomes	Key Notes
Bisphenols	Canned foods Bottled beverages Plastic food containers	Detected in maternal urine Serum, amniotic fluid; crosses placenta	Altered neurobehavior, changes in birth weight, metabolic alterations [49]	BPA restrictions led to substitution with BPS/BPF, which may have similar effects [50]
Phthalates	Packaged and processed foods High-fat foods (dairy, meat, oils)	Maternal urinary metabolites widely detected	Altered reproductive development, increased asthma and wheeze risk, endocrine disruption [34,35]	Ubiquitous in food packaging; migrate easily into food [35]
Pesticides	Fruits, Vegetables, Grains	Detected in maternal blood and urine	Reduced birth weight, impaired neurocognitive development, endocrine dysfunction [39,40]	Exposure continues despite regulatory controls; cumulative effects possible [41]
Persistent Organic Pollutants	Fish, meat Dairy (bioaccumulation in fat)	Long biological half-life; detected in maternal blood, placenta, and breast milk	Thyroid disruption, immunotoxicity, metabolic disorders (obesity, diabetes) [47]	Persistent in environment; transgenerational effects of concern [47]

**Table 2:** Effects of prenatal exposure to EDC on the health of the offspring, including the systems, mechanisms, and results that are impacted.

System/Outcome	EDCs Implicated	Mechanisms	Observed Effects in Offspring
Neurodevelopment [73,74]	Bisphenols Phthalates	Hormonal disruption, OS Epigenetic reprogramming	ADHD, ASD-like traits Cognitive decline Lower IQ Altered synapse formation
Metabolic Health [75]	BPA Phthalates POPs	Epigenetic changes Endocrine signaling disruption Pancreatic β-cell alterations	Obesity Insulin resistance Metabolic syndrome
Reproductive Outcomes [76,77]	BPA Phthalates	Hormonal disruption Epigenetic alterations	Males: lower sperm count, disrupted testicular architecture, reduced motility Females: altered ovarian follicles, disrupted estrous cycles, hormonal changes
Immune Function [78]	POPs Bisphenols	OS Epigenetic modifications Cytokine and immune cell dysregulation	Weaker vaccine response Higher susceptibility to infections Increased allergies, eczema, asthma

**Table 3:** Public health strategies to reduce prenatal dietary exposure to EDCs.

Strategy	Target	Description/Examples	Potential Benefits
Regulatory Measures [118,121]	Governments, Regulatory agencies	Restrict use of bisphenols, phthalates, and other harmful chemicals in food packaging; enforce pesticide residue limits; monitor POPs in the food supply	Reduces maternal and fetal exposure; ensures safer food products
Public Education [122]	Pregnant women, General public	Inform about dietary sources of EDCs; promote consumption of fresh/minimally processed foods; avoid heating food in plastic containers	Empowers consumers to make safer dietary choices; lowers cumulative exposure
Clinical Guidance [4]	Healthcare providers	Integrate EDC risk awareness into prenatal care; provide practical advice on minimizing exposure	Enhances patient knowledge; supports informed decision-making during pregnancy
Research and Monitoring [123]	Scientists, Public health agencies	Conduct studies on EDC exposure, mixture effects, and long-term outcomes; develop exposure guidelines	Informs policy and prevention strategies; fills knowledge gaps

## Data Availability

No new data were created or analyzed in this study. Data sharing is not applicable to this article.

## Abbreviations

The following abbreviations are used in this manuscript:

EDCs	Endocrine-Disrupting Chemicals
POPs	Persistent Organic Pollutants
OS	Oxidative Stress
PCBs	Polychlorinated Bisphenols
BPA	Bisphenol A
BPS	Bisphenol S
BPF	Bisphenol F
ROS	Reactive Oxygen Species
ADHD	Attention-deficit/hyperactivity disorder

## References

[1] Davis E.P., Narayan A.J. (2020). Pregnancy as a period of risk, adaptation, and resilience for mothers and infants. Dev. Psychopathol..

[2] Nobile S., Di Sipio Morgia C., Vento G. (2022). Perinatal Origins of Adult Disease and Opportunities for Health Promotion: A Narrative Review. J. Pers. Med..

[3] Lacagnina S. (2020). The Developmental Origins of Health and Disease (DOHaD). Am. J. Lifestyle Med..

[4] Braun J.M. (2017). Early-life exposure to EDCs: Role in childhood obesity and neurodevelopment. Nat. Rev. Endocrinol..

[5] Cernigliaro F., Santangelo A., Nardello R., Lo Cascio S., D’Agostino S., Correnti E., Marchese F., Pitino R., Valdese S., Rizzo C. (2024). Prenatal Nutritional Factors and Neurodevelopmental Disorders: A Narrative Review. Life.

[6] Segal T.R., Giudice L.C. (2019). Before the beginning: Environmental exposures and reproductive and obstetrical outcomes. Fertil. Steril..

[7] Toledano J.M., Puche-Juarez M., Moreno-Fernandez J., Gonzalez-Palacios P., Rivas A., Ochoa J.J., Diaz-Castro J. (2024). Implications of Prenatal Exposure to Endocrine-Disrupting Chemicals in Offspring Development: A Narrative Review. Nutrients.

[8] Karakoltzidis A., Karakitsios S.P., Gabriel C., Sarigiannis D.A. (2025). Integrated PBPK modelling for PFOA exposure and risk assessment. Environ. Res..

[9] Jaskulak M., Zimowska M., Rolbiecka M., Zorena K. (2025). Understanding the role of endocrine disrupting chemicals as environmental obesogens in the obesity epidemic: A comprehensive overview of epidemiological studies between 2014 and 2024. Ecotoxicol. Environ. Saf..

[10] Martyniuk C.J., Martínez R., Navarro-Martín L., Kamstra J.H., Schwendt A., Reynaud S., Chalifour L. (2022). Emerging concepts and opportunities for endocrine disruptor screening of the non-EATS modalities. Environ. Res..

[11] Merrill A.K., Sobolewski M., Susiarjo M. (2023). Exposure to endocrine disrupting chemicals impacts immunological and metabolic status of women during pregnancy. Mol. Cell Endocrinol..

[12] Gupta R.K., Pipliya S., Karunanithi S., Eswaran U.G.M., Kumar S., Mandliya S., Srivastav P.P., Suthar T., Shaikh A.M., Harsányi E. (2024). Migration of Chemical Compounds from Packaging Materials into Packaged Foods: Interaction, Mechanism, Assessment, and Regulations. Foods.

[13] Raheem D., Trovò M., Carmona Mora C., Vassent C. (2025). Persistent Organic Pollutants’ Threats and Impacts on Food Safety in the Polar Regions—A Concise Review. Pollutants.

[14] Đokić M., Nekić T., Varenina I., Varga I., Solomun Kolanović B., Sedak M., Čalopek B., Vratarić D., Bilandžić N. (2024). Pesticides and Polychlorinated Biphenyls in Milk and Dairy Products in Croatia: A Health Risk Assessment. Foods.

[15] Yan Y., Guo F., Liu K., Ding R., Wang Y. (2023). The effect of endocrine-disrupting chemicals on placental development. Front. Endocrinol..

[16] Rabotnick M.H., Ehlinger J., Haidari A., Goodrich J.M. (2023). Prenatal exposures to endocrine disrupting chemicals: The role of multi-omics in understanding toxicity. Mol. Cell Endocrinol..

[17] Moosavi A., Motevalizadeh Ardekani A. (2016). Role of Epigenetics in Biology and Human Diseases. Iran. Biomed. J..

[18] Gapp K., Von Ziegler L., Tweedie-Cullen R.Y., Mansuy I.M. (2014). Early life epigenetic programming and transmission of stress-induced traits in mammals: How and when can environmental factors influence traits and their transgenerational inheritance?. BioEssays.

[19] Peivasteh-Roudsari L., Barzegar-Bafrouei R., Sharifi K.A., Azimisalim S., Karami M., Abedinzadeh S., Asadinezhad S., Tajdar-Oranj B., Mahdavi V., Alizadeh A.M. (2023). Origin, dietary exposure, and toxicity of endocrine-disrupting food chemical contaminants: A comprehensive review. Heliyon.

[20] Huang R., Zhou G., Cai J., Cao C., Zhu Z., Wu Q., Zhang F., Ding Y. (2025). Maternal consumption of urbanized diet compromises early-life health in association with gut microbiota. Gut Microbes.

[21] Lazarevic N., Barnett A.G., Sly P.D., Knibbs L.D. (2019). Statistical Methodology in Studies of Prenatal Exposure to Mixtures of Endocrine-Disrupting Chemicals: A Review of Existing Approaches and New Alternatives. Environ. Health Perspect..

[22] Lowe K., Dawson J., Phillips K., Minucci J., Wambaugh J.F., Qian H., Ramanarayanan T., Egeghy P., Ingle B., Brunner R. (2021). Incorporating human exposure information in a weight of evidence approach to inform design of repeated dose animal studies. Regul. Toxicol. Pharmacol. RTP.

[23] Puche-Juarez M., Toledano J.M., Moreno-Fernandez J., Gálvez-Ontiveros Y., Rivas A., Diaz-Castro J., Ochoa J.J. (2023). The Role of Endocrine Disrupting Chemicals in Gestation and Pregnancy Outcomes. Nutrients.

[24] Hoepner L.A. (2019). Bisphenol a: A narrative review of prenatal exposure effects on adipogenesis and childhood obesity via peroxisome proliferator-activated receptor gamma. Environ. Res..

[25] Manzoor M.F., Tariq T., Fatima B., Sahar A., Tariq F., Munir S., Khan S., Ranjha M.M.A.N., Sameen A., Zeng X.-A. (2022). An insight into bisphenol A, food exposure and its adverse effects on health: A review. Front. Nutr..

[26] Harnett K.G., Chin A., Schuh S.M. (2021). BPA and BPA alternatives BPS, BPAF, and TMBPF, induce cytotoxicity and apoptosis in rat and human stem cells. Ecotoxicol. Environ. Saf..

[27] Hajjar R., Hatoum S., Mattar S., Moawad G., Ayoubi J.M., Feki A., Ghulmiyyah L. (2024). Endocrine Disruptors in Pregnancy: Effects on Mothers and Fetuses-A Review. J. Clin. Med..

[28] Beck A.L., Bräuner E.V., Uldbjerg C.S., Lim Y.H., Boye H., Frederiksen H., Andersson A.-M., Jensen T.K. (2025). Maternal urinary concentrations of bisphenol A during pregnancy and birth size in children from the Odense Child Cohort. Environ. Health Glob. Access Sci. Source.

[29] Wiklund L., Beronius A. (2022). Systematic evaluation of the evidence for identification of endocrine disrupting properties of Bisphenol F. Toxicology.

[30] Reininger N., Oehlmann J. (2024). Regrettable substitution? Comparative study of the effect profile of bisphenol A and eleven analogues in an in vitro test battery. Environ. Sci. Eur..

[31] Giuliani A., Zuccarini M., Cichelli A., Khan H., Reale M. (2020). Critical Review on the Presence of Phthalates in Food and Evidence of Their Biological Impact. Int. J. Environ. Res. Public Health.

[32] Tanzer M., Boissiere-O’Neill T., Sly P.D., Vilcins D. (2025). Phthalates, bisphenols and per-and polyfluoroalkyl substances migration from food packaging into food: A systematic review. Rev. Environ. Health.

[33] Almeida-Toledano L., Navarro-Tapia E., Sebastiani G., Ferrero-Martínez S., Ferrer-Aguilar P., García-Algar Ó., Andreu-Fernández V., Gómez-Roig M.D. (2024). Effect of prenatal phthalate exposure on fetal development and maternal/neonatal health consequences: A systematic review. Sci. Total Environ..

[34] Jøhnk C., Høst A., Husby S., Schoeters G., Timmermann C.A.G., Kyhl H.B., Beck I.H., Andersson A.-M., Frederiksen H., Jensen T.K. (2020). Maternal phthalate exposure and asthma, rhinitis and eczema in 552 children aged 5 years; a prospective cohort study. Environ. Health.

[35] Boissiere-O’Neill T., Lazarevic N., Sly P.D., Ponsonby A.L., Chen A., Azad M.B., Braun J.M., Brook J.R., Burgner D., Lanphear B.P. (2025). Phthalates and Bisphenols Early-Life Exposure, and Childhood Allergic Conditions: A Pooled Analysis of Cohort Studies. J. Expo. Sci. Environ. Epidemiol..

[36] Hlisníková H., Petrovičová I., Kolena B., Šidlovská M., Sirotkin A. (2020). Effects and Mechanisms of Phthalates’ Action on Reproductive Processes and Reproductive Health: A Literature Review. Int. J. Environ. Res. Public Health.

[37] Ahmad M.F., Ahmad F.A., Alsayegh A.A., Zeyaullah M., AlShahrani A.M., Muzammil K., Saati A.A., Wahab S., Elbendary E.Y., Kambal N. (2024). Pesticides impacts on human health and the environment with their mechanisms of action and possible countermeasures. Heliyon.

[38] Pathak V.M., Verma V.K., Rawat B.S., Kaur B., Babu N., Sharma A., Dewali S., Yadav M., Kumari R., Singh S. (2022). Current status of pesticide effects on environment, human health and it’s eco-friendly management as bioremediation: A comprehensive review. Front. Microbiol..

[39] Peterson B.S., Delavari S., Bansal R., Sawardekar S., Gupte C., Andrews H., Hoepner L.A., Garcia W., Perera F., Rauh V. (2025). Brain Abnormalities in Children Exposed Prenatally to the Pesticide Chlorpyrifos. JAMA Neurol..

[40] Sagiv S.K., Baker J.M., Rauch S., Gao Y., Gunier R.B., Mora A.M., Kogut K., Bradman A., Eskenazi B., Reiss A.L. (2024). Prenatal and childhood exposure to organophosphate pesticides and functional brain imaging in young adults. Environ. Res..

[41] Liu J., Schelar E. (2012). Pesticide exposure and child neurodevelopment: Summary and implications. Workplace Health Saf..

[42] Sittiwang S., Nimmapirat P., Suttiwan P., Promduang W., Chaikittipornlert N., Wouldes T., Prapamontol T., Naksen W., Promkam N., Pingwong S. (2022). The relationship between prenatal exposure to organophosphate insecticides and neurodevelopmental integrity of infants at 5-weeks of age. Front. Epidemiol..

[43] Guo W., Pan B., Sakkiah S., Yavas G., Ge W., Zou W., Tong W., Hong H. (2019). Persistent Organic Pollutants in Food: Contamination Sources, Health Effects and Detection Methods. Int. J. Environ. Res. Public Health.

[44] Maruyama K., Oshima T., Ohyama K. (2010). Exposure to exogenous estrogen through intake of commercial milk produced from pregnant cows. Pediatr. Int. Off J. Jpn. Pediatr. Soc..

[45] Lauritzen H.B., Larose T.L., Øien T., Sandanger T.M., Odland J.Ø., van de Bor M., Jacobsen G.W. (2018). Prenatal exposure to persistent organic pollutants and child overweight/obesity at 5-year follow-up: A prospective cohort study. Environ. Health Glob. Access Sci. Source.

[46] Stratakis N., Rock S., La Merrill M.A., Saez M., Robinson O., Fecht D., Vrijheid M., Valvi D., Conti D.V., McConnell R. (2022). Prenatal exposure to persistent organic pollutants and childhood obesity: A systematic review and meta-analysis of human studies. Obes. Rev. Off J. Int. Assoc. Study Obes..

[47] Ashley-Martin J., Levy A.R., Arbuckle T.E., Platt R.W., Marshall J.S., Dodds L. (2015). Maternal exposure to metals and persistent pollutants and cord blood immune system biomarkers. Environ. Health Glob. Access Sci. Source.

[48] Hu L., Luo D., Wang L., Yu M., Zhao S., Wang Y., Mei S., Zhang G. (2021). Levels and profiles of persistent organic pollutants in breast milk in China and their potential health risks to breastfed infants: A review. Sci. Total Environ..

[49] Algonaiman R., Almutairi A.S., Al Zhrani M.M., Barakat H. (2023). Effects of Prenatal Exposure to Bisphenol A Substitutes, Bisphenol S and Bisphenol F, on Offspring’s Health: Evidence from Epidemiological and Experimental Studies. Biomolecules.

[50] Costa H.E., Medeiros I., Mariana M., Cairrao E. (2025). Maternal–Foetal Effects of Exposure to Bisphenol A: Outcomes and Long-Term Consequences. Appl. Sci..

[51] Yang Z., Zhang J., Wang M., Wang X., Liu H., Zhang F., Fan H. (2024). Prenatal endocrine-disrupting chemicals exposure and impact on offspring neurodevelopment: A systematic review and meta-analysis. Neurotoxicology.

[52] Kahn L.G., Philippat C., Nakayama S.F., Slama R., Trasande L. (2020). Endocrine-disrupting chemicals: Implications for human health. Lancet Diabetes Endocrinol..

[53] Predieri B., Iughetti L., Bernasconi S., Street M.E. (2022). Endocrine Disrupting Chemicals’ Effects in Children: What We Know and What We Need to Learn?. Int. J. Mol. Sci..

[54] Alavian-Ghavanini A., Rüegg J. (2018). Understanding Epigenetic Effects of Endocrine Disrupting Chemicals: From Mechanisms to Novel Test Methods. Basic Clin. Pharmacol. Toxicol..

[55] Kunysz M., Mora-Janiszewska O., Darmochwał-Kolarz D. (2021). Epigenetic Modifications Associated with Exposure to Endocrine Disrupting Chemicals in Patients with Gestational Diabetes Mellitus. Int. J. Mol. Sci..

[56] Kowalczyk A., Wrzecińska M., Czerniawska-Piątkowska E., Araújo J.P., Cwynar P. (2022). Molecular consequences of the exposure to toxic substances for the endocrine system of females. Biomed. Pharmacother..

[57] Martini M., Corces V.G., Rissman E.F. (2020). Mini-review: Epigenetic mechanisms that promote transgenerational actions of endocrine disrupting chemicals: Applications to behavioral neuroendocrinology. Horm. Behav..

[58] Pan J., Liu P., Yu X., Zhang Z., Liu J. (2024). The adverse role of endocrine disrupting chemicals in the reproductive system. Front. Endocrinol..

[59] Miranda A., Sousa N. (2018). Maternal hormonal milieu influence on fetal brain development. Brain Behav..

[60] Zubeldia-Brenner L., Roselli C.E., Recabarren S.E., Gonzalez Deniselle M.C., Lara H.E. (2016). Developmental and Functional Effects of Steroid Hormones on the Neuroendocrine Axis and Spinal Cord. J. Neuroendocrinol..

[61] Rolfo A., Nuzzo A.M., De Amicis R., Moretti L., Bertoli S., Leone A. (2020). Fetal-Maternal Exposure to Endocrine Disruptors: Correlation with Diet Intake and Pregnancy Outcomes. Nutrients.

[62] Lite C., Raja G.L., Juliet M., Sridhar V.V., Subhashree K.D., Kumar P., Chakraborty P., Arockiaraj J. (2022). In utero exposure to endocrine-disrupting chemicals, maternal factors and alterations in the epigenetic landscape underlying later-life health effects. Environ. Toxicol. Pharmacol..

[63] Aouache R., Biquard L., Vaiman D., Miralles F. (2018). Oxidative Stress in Preeclampsia and Placental Diseases. Int. J. Mol. Sci..

[64] Meli R., Monnolo A., Annunziata C., Pirozzi C., Ferrante M.C. (2020). Oxidative Stress and BPA Toxicity: An Antioxidant Approach for Male and Female Reproductive Dysfunction. Antioxidants.

[65] Aris I.M., Fleisch A.F., Oken E. (2018). Developmental Origins of Disease: Emerging Prenatal Risk Factors and Future Disease Risk. Curr. Epidemiol. Rep..

[66] Lu Z., Guo Y., Xu D., Xiao H., Dai Y., Liu K., Chen L., Wang H. (2023). Developmental toxicity and programming alterations of multiple organs in offspring induced by medication during pregnancy. Acta Pharm. Sin. B.

[67] Sferruzzi-Perri A.N., Lopez-Tello J., Salazar-Petres E. (2023). Placental adaptations supporting fetal growth during normal and adverse gestational environments. Exp. Physiol..

[68] Dumolt J.H., Powell T.L., Jansson T. (2021). Placental Function and the Development of Fetal Overgrowth and Fetal Growth Restriction. Obstet. Gynecol. Clin. N. Am..

[69] Schjenken J.E., Green E.S., Overduin T.S., Mah C.Y., Russell D.L., Robertson S.A. (2021). Endocrine Disruptor Compounds-A Cause of Impaired Immune Tolerance Driving Inflammatory Disorders of Pregnancy?. Front. Endocrinol..

[70] Street M.E., Bernasconi S. (2020). Endocrine-Disrupting Chemicals in Human Fetal Growth. Int. J. Mol. Sci..

[71] Di Pietro G., Forcucci F., Chiarelli F. (2023). Endocrine Disruptor Chemicals and Children’s Health. Int. J. Mol. Sci..

[72] Kumar M., Sarma D.K., Shubham S., Kumawat M., Verma V., Prakash A., Tiwari R. (2020). Environmental Endocrine-Disrupting Chemical Exposure: Role in Non-Communicable Diseases. Front. Public Health.

[73] Minatoya M., Kishi R. (2021). A Review of Recent Studies on Bisphenol A and Phthalate Exposures and Child Neurodevelopment. Int. J. Environ. Res. Public Health.

[74] Stein T.P., Schluter M.D., Steer R.A., Ming X. (2023). Bisphenol-A and phthalate metabolism in children with neurodevelopmental disorders. PLoS ONE.

[75] Symeonides C., Aromataris E., Mulders Y., Dizon J., Stern C., Barker T.H., Whitehorn A., Pollock D., Marin T., Dunlop S. (2024). An Umbrella Review of Meta-Analyses Evaluating Associations between Human Health and Exposure to Major Classes of Plastic-Associated Chemicals. Ann. Glob. Health.

[76] Paoli D., Pallotti F., Dima A.P., Albani E., Alviggi C., Causio F., Dioguardi C.C., Conforti A., Ciriminna R., Fabozzi G. (2020). Phthalates and Bisphenol A: Presence in Blood Serum and Follicular Fluid of Italian Women Undergoing Assisted Reproduction Techniques. Toxics.

[77] Tzouma Z., Dourou P., Diamanti A., Harizopoulou V., Papalexis P., Karampas G., Liepinaitienė A., Dėdelė A., Sarantaki A. (2025). Associations Between Endocrine-Disrupting Chemical Exposure and Fertility Outcomes: A Decade of Human Epidemiological Evidence. Life.

[78] Berghuis S.A., Bos A.F., Sauer P.J.J., Roze E. (2015). Developmental neurotoxicity of persistent organic pollutants: An update on childhood outcome. Arch. Toxicol..

[79] O’Shaughnessy K.L., Fischer F., Zenclussen A.C. (2021). Perinatal exposure to endocrine disrupting chemicals and neurodevelopment: How articles of daily use influence the development of our children. Best Pract. Res. Clin. Endocrinol. Metab..

[80] Zoppé H., Xavier J., Dupuis A., Migeot V., Bioulac S., Hary R., Bonnet-Brilhault F., Albouy M. (2024). Is exposure to Bisphenol A associated with Attention-deficit hyperactivity disorder (ADHD) and associated executive or behavioral problems in children? A comprehensive systematic review. Neurosci. Biobehav. Rev..

[81] Santoro A., Chianese R., Troisi J., Richards S., Nori S.L., Fasano S., Guida M., Plunk E., Viggiano A., Pierantoni R. (2019). Neuro-toxic and Reproductive Effects of BPA. Curr. Neuropharmacol..

[82] Palanza P., Gioiosa L., Vom Saal F.S., Parmigiani S. (2008). Effects of developmental exposure to bisphenol A on brain and behavior in mice. Environ. Res..

[83] Kajta M., Wójtowicz A.K. (2013). Impact of endocrine-disrupting chemicals on neural development and the onset of neurological disorders. Pharmacol. Rep..

[84] Hojo R., Zareba G., Kai J.W., Baggs R.B., Weiss B. (2006). Sex-specific alterations of cerebral cortical cell size in rats exposed prenatally to dioxin. J. Appl. Toxicol..

[85] Rebuli M.E., Speen A.M., Clapp P.W., Jaspers I. (2017). Novel applications for a noninvasive sampling method of the nasal mucosa. Am. J. Physiol. Lung Cell Mol. Physiol..

[86] Swaab D.F., Wolff S.E.C., Bao A.M. (2021). Sexual differentiation of the human hypothalamus: Relationship to gender identity and sexual orientation. Handb. Clin. Neurol..

[87] Güil-Oumrait N., Stratakis N., Maitre L., Anguita-Ruiz A., Urquiza J., Fabbri L., Basagaña X., Heude B., Haug L.S., Sakhi A.K. (2024). Prenatal Exposure to Chemical Mixtures and Metabolic Syndrome Risk in Children. JAMA Netw. Open..

[88] Celik M.N., Yesildemir O. (2025). Endocrine Disruptors in Child Obesity and Related Disorders: Early Critical Windows of Exposure. Curr. Nutr. Rep..

[89] Wu Y.L., Lin Z.J., Li C.C., Lin X., Shan S.K., Guo B., Zheng M.-H., Li F., Yuan L.-Q., Li Z.-H. (2023). Epigenetic regulation in metabolic diseases: Mechanisms and advances in clinical study. Signal Transduct. Target. Ther..

[90] Quilichini E., Haumaitre C. (2015). Implication of epigenetics in pancreas development and disease. Best Pract. Res. Clin. Endocrinol. Metab..

[91] Xu T., Dong C., Shao J., Huo C., Chen Z., Shi Z., Yao T., Gu C., Wei W., Rui D. (2024). Global burden of maternal disorders attributable to malnutrition from 1990 to 2019 and predictions to 2035: Worsening or improving?. Front. Nutr..

[92] Price S.A.L., Koye D.N., Lewin A., Nankervis A., Kane S.C. (2025). Maternal Metabolic Health and Mother and Baby Health Outcomes (MAMBO): Protocol of a Prospective Observational Study. JMIR Res. Protoc..

[93] Perez-Escamilla R., Bermudez O., Buccini G.S., Kumanyika S., Lutter C.K., Monsivais P., Victora C. (2018). Nutrition disparities and the global burden of malnutrition. BMJ.

[94] Salami E.A., Rotimi O.A. (2024). The impact of Bisphenol-A on human reproductive health. Toxicol. Rep..

[95] Wang L., Ma X., Liu J. (2025). Adverse Effects of Pesticides on the Ovary: Evidence from Epidemiological and Toxicological Studies. Environ. Health.

[96] Zhou C., Gao L., Flaws J.A. (2017). Prenatal exposure to an environmentally relevant phthalate mixture disrupts reproduction in F1 female mice. Toxicol. Appl. Pharmacol..

[97] Street M.E., Angelini S., Bernasconi S., Burgio E., Cassio A., Catellani C., Cirillo F., Deodati A., Fabbrizi E., Fanos V. (2018). Current Knowledge on Endocrine Disrupting Chemicals (EDCs) from Animal Biology to Humans, from Pregnancy to Adulthood: Highlights from a National Italian Meeting. Int. J. Mol. Sci..

[98] Demir A., Aydin A., Büyükgebiz A. (2025). Thematic Review of Endocrine Disruptors and Their Role in Shaping Pubertal Timing. Children.

[99] Emokpae M.A., Brown S.I. (2021). Effects of lifestyle factors on fertility: Practical recommendations for modification. Reprod. Fertil..

[100] Sharma R., Biedenharn K.R., Fedor J.M., Agarwal A. (2013). Lifestyle factors and reproductive health: Taking control of your fertility. Reprod. Biol. Endocrinol..

[101] Ghozal M., Kadawathagedara M., Delvert R., Divaret-Chauveau A., Raherison C., Varraso R., Bédard A., Crépet A., Sirot V., Charles M.A. (2024). Prenatal dietary exposure to mixtures of chemicals is associated with allergy or respiratory diseases in children in the ELFE nationwide cohort. Environ. Health.

[102] Moustakli E., Potiris A., Grigoriadis T., Zikopoulos A., Drakaki E., Zouganeli I., Theofanakis C., Gerede A., Zachariou A., Domali E. (2025). Unraveling the Core of Endometriosis: The Impact of Endocrine Disruptors. Int. J. Mol. Sci..

[103] Schug T.T., Janesick A., Blumberg B., Heindel J.J. (2011). Endocrine disrupting chemicals and disease susceptibility. J. Steroid Biochem. Mol. Biol..

[104] Liang Y., Lu Q., Chen M., Zhao X., Chu C., Zhang C., Yuan J., Liu H., Lash G.E. (2025). Impact of endocrine disrupting chemicals (EDCs) on epigenetic regulation in the uterus: A narrative review. Reprod. Biol. Endocrinol..

[105] Marques A.H., O’Connor T.G., Roth C., Susser E., Bjørke-Monsen A.L. (2013). The influence of maternal prenatal and early childhood nutrition and maternal prenatal stress on offspring immune system development and neurodevelopmental disorders. Front. Neurosci..

[106] Fox R., Akinboro S., Kędzia A., Niechciał E. (2025). The Effects of Maternal Endocrinopathies and Exposure to Endocrine Disruptors During Pregnancy on the Fetus and Newborn. Biomedicines.

[107] Lenters V., Sugeng E., Van Duursen M.B.M. Safeguarding Women’s Health Against Endocrine Disrupting Chemicals: Paving the Way to Successful Health Strategies. https://rgdoi.net/10.13140/RG.2.2.27889.29281.

[108] Ahmed S.K., Mohammed R.A. (2025). Obesity: Prevalence, causes, consequences, management, preventive strategies and future research directions. Metab. Open.

[109] Seref N., Cufaoglu G. (2025). Food Packaging and Chemical Migration: A Food Safety Perspective. J. Food Sci..

[110] Li X., Shen X., Jiang W., Xi Y., Li S. (2024). Comprehensive review of emerging contaminants: Detection technologies, environmental impact, and management strategies. Ecotoxicol. Environ. Saf..

[111] Okman E., Yalçın S.S. (2024). Awareness and Knowledge of Endocrine-Disrupting Chemicals Among Pregnant Women and New Mothers: A Cross-Sectional Survey Study. Toxics.

[112] Rouillon S., El Ouazzani H., Hardouin J.B., Enjalbert L., Rabouan S., Migeot V., Albouy-Llaty M. (2020). How to Educate Pregnant Women about Endocrine Disruptors?. Int. J. Environ. Res. Public Health.

[113] Chinglenthoiba C., Lani M.N., Anuar S.T., Amesho K.T.T., K L P., Santos J.H. (2025). Microplastics in food packaging: Analytical methods, health risks, and sustainable alternatives. J. Hazard. Mater. Adv..

[114] Mafe A.N., Edo G.I., Makia R.S., Joshua O.A., Akpoghelie P.O., Gaaz T.S., Jikah A.N., Yousif E., Isoje E.F., Igbuku U.A. (2024). A review on food spoilage mechanisms, food borne diseases and commercial aspects of food preservation and processing. Food Chem. Adv..

[115] Meyyazhagan A., Kuchi Bhotla H., Tsibizova V., Pappuswamy M., Chaudhary A., Arumugam V.A., Al Qasem M., Di Renzo G.C. (2023). Nutrition paves the way to environmental toxicants and influences fetal development during pregnancy. Best Pract. Res. Clin. Obstet. Gynaecol..

[116] Brink L.R., Bender T.M., Davies R., Luo H., Miketinas D., Shah N., Loveridge N., Gross G., Fawkes N. (2022). Optimizing Maternal Nutrition: The Importance of a Tailored Approach. Curr. Dev. Nutr..

[117] Alehegn M.A., Fanta T.K., Ayalew A.F. (2021). Exploring maternal nutrition counseling provided by health professionals during antenatal care follow-up: A qualitative study in Addis Ababa, Ethiopia-2019. BMC Nutr..

[118] Duh-Leong C., Maffini M.V., Kassotis C.D., Vandenberg L.N., Trasande L. (2023). The regulation of endocrine-disrupting chemicals to minimize their impact on health. Nat. Rev. Endocrinol..

[119] Oyovwi M.O., Oyelami J.O., Babawale K.H., Ben-Azu B. (2025). Preventive antioxidant strategies to reduce the effects of phthalate exposure on reproductive health. Discov. Environ..

[120] Haggerty D.K., Upson K., Pacyga D.C., Franko J.E., Braun J.M., Strakovsky R.S. (2021). REPRODUCTIVE TOXICOLOGY: Pregnancy exposure to endocrine disrupting chemicals: Implications for women’s health. Reprod. Camb. Engl..

[121] Trela-Kobędza E., Ajduk A. (2025). The impact of bisphenol A and its analogs on female reproductive health. Reprod. Biol..

[122] Martin L., Zhang Y., First O., Mustieles V., Dodson R., Rosa G., Coburn-Sanderson A., Adams C.D., Messerlian C. (2022). Lifestyle interventions to reduce endocrine-disrupting phthalate and phenol exposures among reproductive age men and women: A review and future steps. Environ. Int..

[123] Meeker J.D. (2012). Exposure to environmental endocrine disruptors and child development. Arch. Pediatr. Adolesc. Med..

